# A Rare THPO Gene Mutation in a Saudi Female Child: A Case Report and Literature Review

**DOI:** 10.7759/cureus.70513

**Published:** 2024-09-30

**Authors:** Badriah G Alasmari, Khalid Elzubair, Ali Alquraishi, Mohammed Adlan, Ali Alabbas, Lina Elzubair, Saeed Al Tala

**Affiliations:** 1 Pediatrics Hematology/Oncology, Armed Forces Hospital Southern Region, Khamis Mushait, SAU; 2 Pediatrics, Armed Forces Hospital Southern Region, Khamis Mushait, SAU; 3 Pediatrics Endocrinology, Armed Forces Hospital Southern Region, Khamis Mushait, SAU; 4 Pediatrics, Najran General Hospital, Najran, SAU; 5 Pathology, Armed Forces Hospital Southern Region, Khamis Mushait, SAU; 6 Pediatrics Genetics, Armed Forces Hospital Southern Region, Khamis Mushait, SAU

**Keywords:** aplastic anemia, bone marrow failure, congenital thrombocytopenia, megakaryocytes, thpo gene

## Abstract

Thrombopoietin (THPO) is a regulator of megakaryopoiesis and thrombopoiesis. Mutation of the *THPO* gene is known to cause congenital amegakaryocytic thrombocytopenia (CAMT2), which is a rare inherited disorder characterized by early infancy thrombocytopenia and absent or decreased megakaryocytes with gradual progression to pancytopenia. We report the case of a Saudi girl who had been asymptomatic until age seven when she was found to have unexplained thrombocytopenia. Whole-genome sequencing detected loss between the genomic coordinates (chr3:184088108-184090520) partially encompassing exon 6 of the *THPO* gene in a homozygous state, which is reported as a new variant.

This report highlights the importance of genetic testing for unexplained persistent hematological abnormalities for early diagnosis, especially in consanguineous populations.

## Introduction

Congenital amegakaryocytic thrombocytopenia type 2 is a rare autosomal recessive disorder caused by mutations in the *THPO* gene encoding for thrombopoietin. The disease usually presents as isolated thrombocytopenia in early infancy with or without physical anomalies and progresses to aplastic anemia and bone marrow failure [1].

Thrombopoietin (THPO), the main regulator of thrombopoiesis and megakaryopoiesis, is encoded at 3q27.1 and includes six coding exons. *THPO* also stimulates the maturation and proliferation of megakaryocytes and promotes the survival, self-renewal, and expansion of hematopoietic stem cells [2,3]. *THPO* interacts with hematopoietic cells via the thrombopoietin receptor (C-mpl). Mutation in the *MPL* gene (on chromosome 1p34.) that encodes this receptor is known to cause congenital amegakaryocytic thrombocytopenia type 1 [4,5], whereas mutation in the *THPO *gene on chromosome 3q27 leads to the loss of function of the gene and is known to cause congenital amegakaryocytic thrombocytopenia type 2 (CAMT2).

In laboratory testing, CAMT2 exhibits normal or decreased serum THPO, as well as hypocellularity of bone marrow with either absent or decreased megakaryocytes. CAMT2 presents in infancy or early childhood with a severe course of the disease or with a mild presentation for those who present later in life [1].

We report the case of a patient who had been following up in the endocrinology clinic for a primary diagnosis of type 1 diabetes mellitus with occasional findings of bruises at insulin injection sites. After presenting to our clinic, following the exclusion of the common causes of late presenting persistent thrombocytopenia, a partial loss of function in exon 6 of the *THPO* gene was detected via whole-genome sequencing.

## Case presentation

The patient was a seven-year-old Saudi girl who was born to first-degree consanguineous parents. She remained healthy until 4.5 years of age when she was diagnosed with type-1 diabetes mellitus, for which she was started on a multiple daily injection regimen by the pediatric endocrine team using both rapid- and long-acting insulins with regular follow-up in the endocrine clinic.

During the follow-up in the endocrine clinic, the patient was noticed to have persistent unexplained thrombocytopenia in the range of 41-90×10^9^/L, and she was referred to the pediatric hematology clinic for further assessment. There was no associated bleeding tendency apart from occasional mild bruises at the site of insulin injection.

Upon further assessment and workup, there was no evidence of acute or chronic infection, malignancy, autoimmune, or connective tissue disorder, and there was no history of use of regular medications apart from insulin. Clinical examinations revealed no abnormalities.

**Figure 1:**
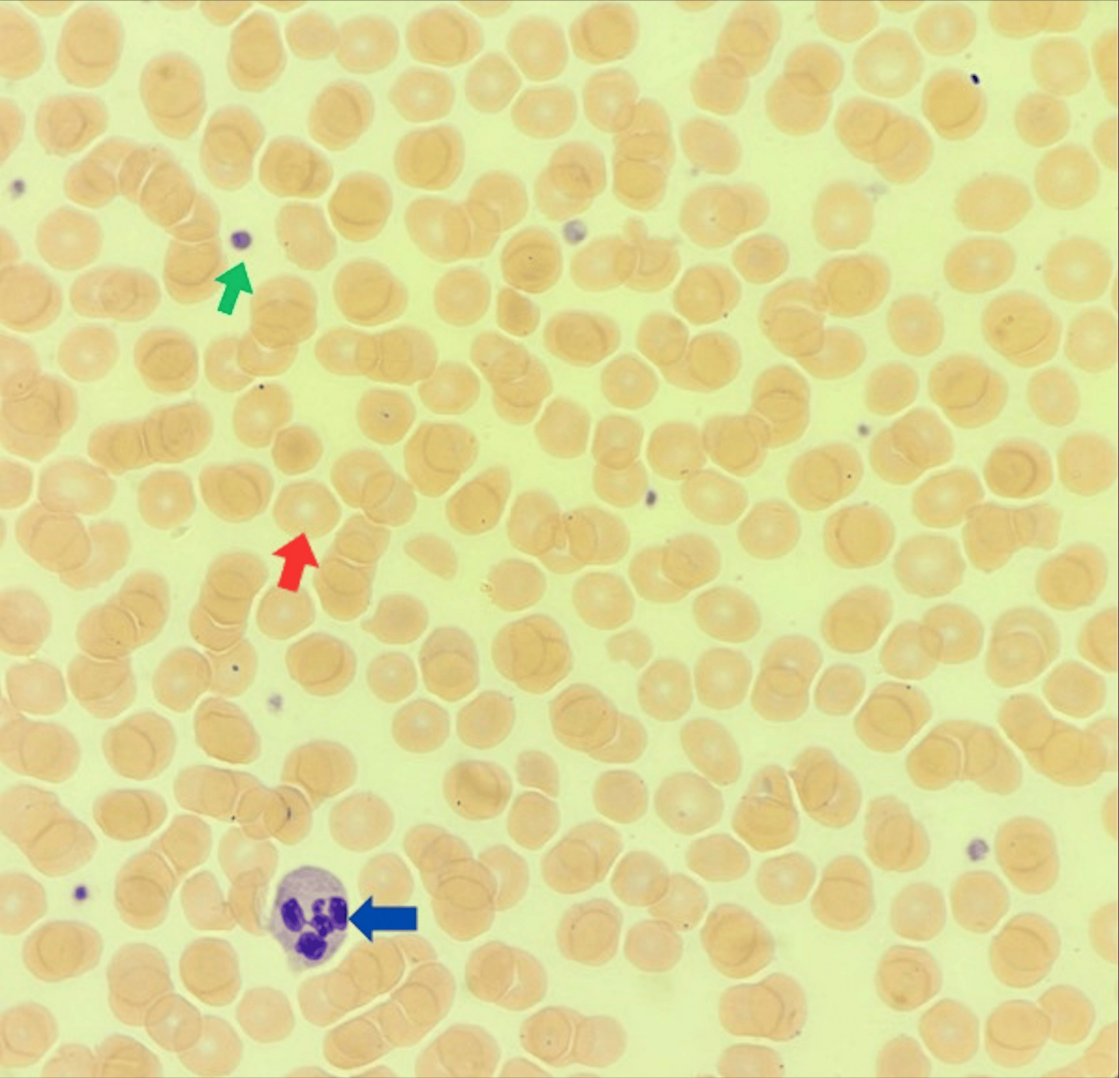
Wright-Giemsa staining of peripheral blood smear (x100) showing normochromic, normocytic red blood cells, adequate neutrophils, no blasts, moderate thrombocytopenia with normal-sized platelets and no platelet clumps Cells are shown with arrows as follows: platelet (green arrow), red blood cells (red arrow), and neutrophil (blue arrow). A manual platelet count of 72x10^9^/L.

**Figure 2:**
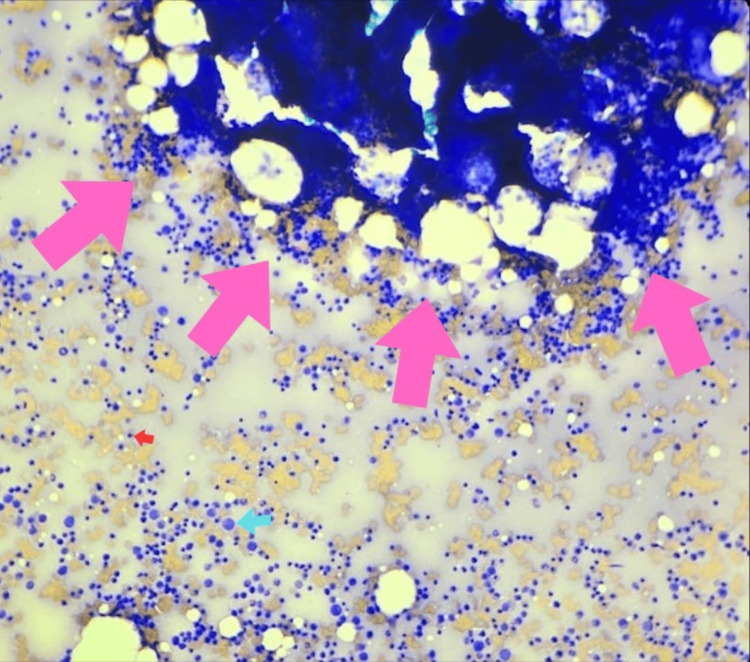
Wright-Giemsa staining of bone marrow aspirate (x20) showing normocellular fragments for age, hyperactive erythropoiesis, active granulopoiesis with ordered maturation, and decreased megakaryopoiesis Cells are shown with arrows as follows: erythroid precursor (red arrow), granulocytic precursor (blue arrow), and bone marrow fragment (pink arrows).

**Figure 3:**
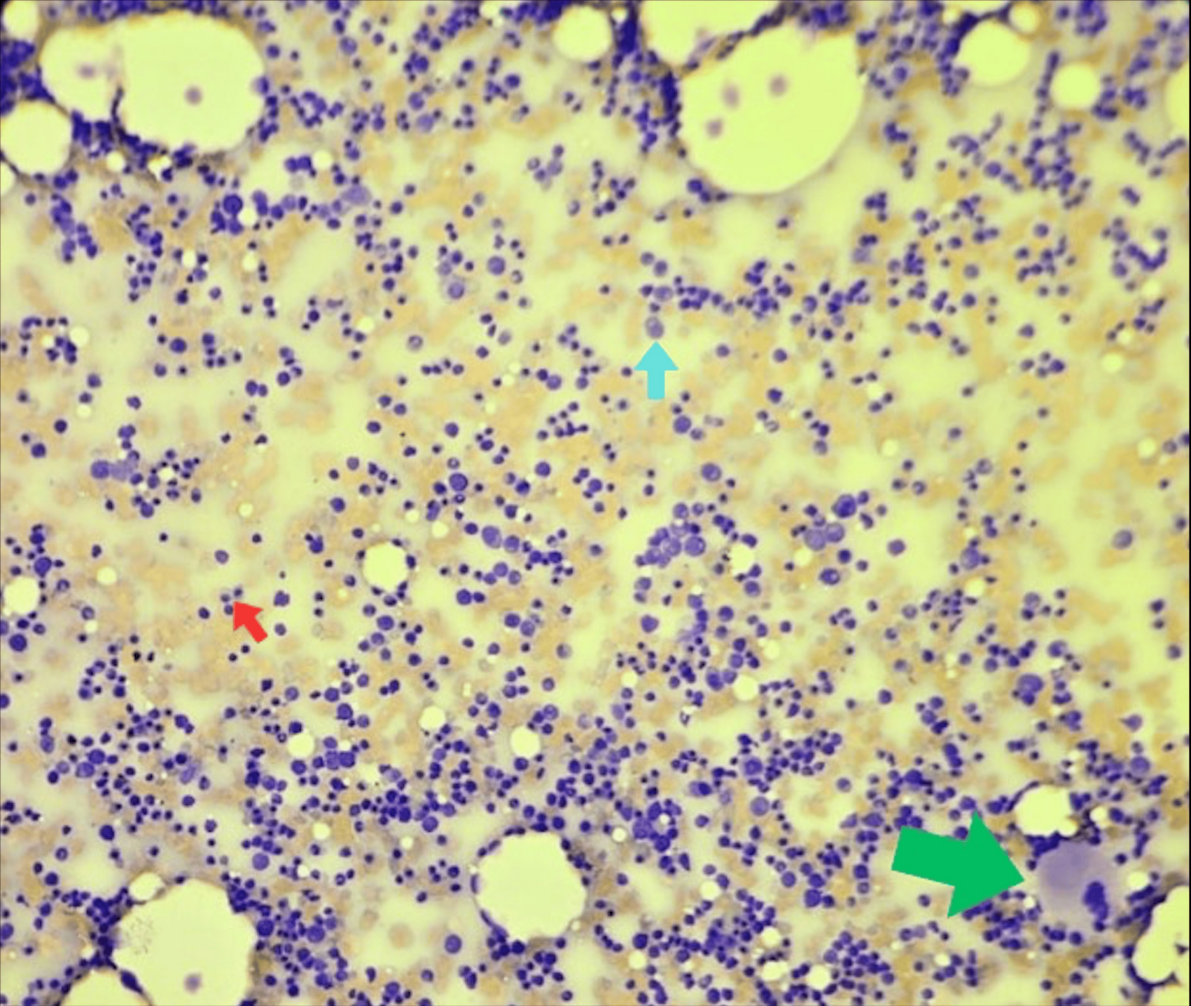
Wright-Giemsa staining of the bone marrow aspirate (x20) showing cellular trail, hyperactive erythropoiesis, active granulopoiesis with ordered maturation, and decreased megakaryopoiesis Cells are shown with arrows as follows: megakaryocyte (green arrow), erythroid precursor (red arrow), and granulocytic precursor (blue arrow).

**Figure 4:**
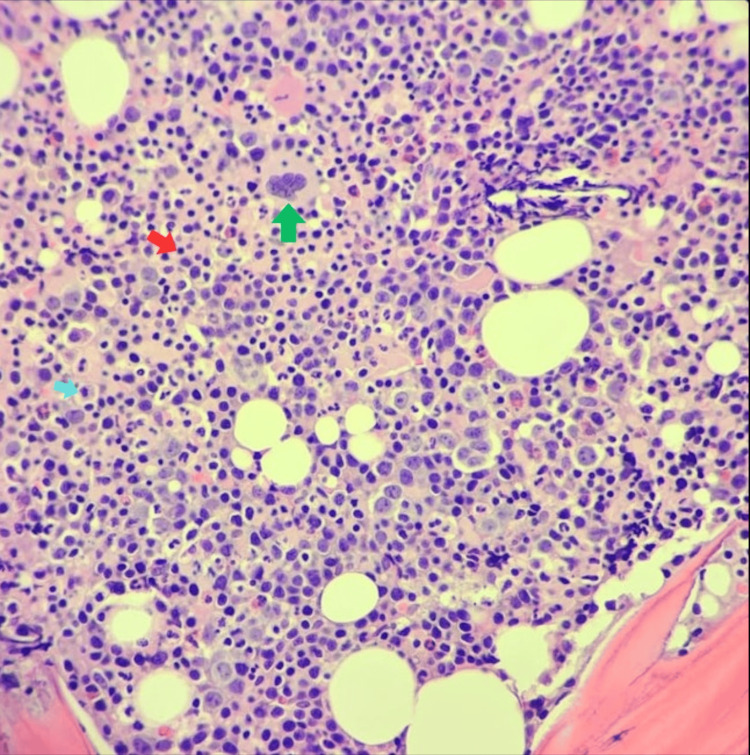
Hematoxylin and eosin staining of trephine biopsy (x40) showing normocellular bone marrow for age with hyperactive erythropoiesis, adequate granulopoiesis with ordered maturation, and decreased megakaryopoiesis with normal maturation

As part of the patient’s workup, a peripheral blood smear showed moderate thrombocytopenia with normal-sized platelets (Figure 1). Bone marrow aspiration revealed decreased megakaryopoiesis (Figures 2, 3). Trephine biopsy revealed normocellular fragment for age, with decreased megakaryopoiesis and normal maturation (Figure 4). Serum THPO level testing was not performed, as it was not available at our clinic.

**Figure 5:**
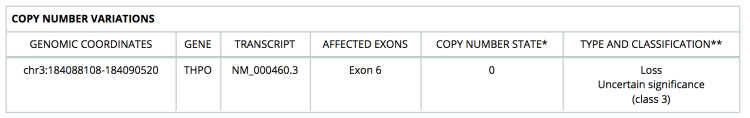
Copy number variations * number of copies of the involved genomic coordinates. ** based on ACMG recommendations. ACMG: American College of Medical Genetics

The patient was followed regularly in the hematology clinic, and she remained asymptomatic with persistent thrombocytopenia and normal hemoglobin level and leucocyte count. Therefore, whole-exome sequencing (WES) was done, as her thrombocytopenia remained unexplained, and there is an increased risk of inherited disorders in Saudi Arabia due to the high rate of consanguineous marriage. WES detected a deletion between genomic coordinates (chr3:+*9184088108-184090520) covering 2412 nucleotides, partially encompassing exon 6, including the stop codon and the 3'UTR of the *THPO* gene, in a homozygous state (Figure 5). Both of the patient’s parents were healthy with normal platelet counts but were found to be heterozygous carriers of this deletion. The mutation was classified as uncertain significance according to the American College of Medical Genetics and Genomics (ACMG)/Association for Molecular Pathology (AMP) ClinGen SVI general recommendations.

Loss of function in the *THPO* gene is a known cause of CAMT2. The patient’s parents were counseled regarding CAMT2, the possibility of progression to full-blown bone marrow failure, and the need to look for signs of anemia, bleeding, and infections. Her other immediate family members were healthy with normal complete blood count readings. There was no family history of any hematological disorder. Currently, the patient continues to exhibit asymptomatic, persistent mild-to-moderate isolated thrombocytopenia under regular follow-up with pediatric hematology and endocrinology.

## Discussion

In this case, the patient had no skeletal anomalies, eczema, recurrent infections, or micro-thrombocytopenia. Therefore, certain causes of thrombocytopenia were excluded such as thrombocytopenia-absent radius and Wiskott-Aldrich syndrome. Workup for infections, malignancy, and autoimmune diseases revealed no explanation for the condition. WES detected a deletion in exon 6 of the *THPO* gene. This deletion would likely result in a significantly altered THPO protein with potentially reduced stability, altered glycosylation, and possibly compromised function. The severity of the effect would depend on the structural and functional role of the lost amino acids and their protein domain.

In 2013, Dasouki et al. first reported the relationship between *THPO* and congenital amegakaryocytic thrombocytopenia in a Micronesian family having autosomal recessive R17C THPO mutation with aplastic anemia in a homozygous state and mild thrombocytopenia in a heterozygous state [6].

In 2017, Seo et al. reported a THPO mutation in five individuals of three unrelated families with severe thrombocytopenia progressing to pancytopenia; one family had a homozygous mutation in THPO R157X, and the other two families had a homozygous mutation in THPO R99W. Both mutations resulted in a low serum THPO level and did not respond to hematopoietic stem cell transplants but showed a good response to THPO receptor agonist therapy [7]. In 2017, Pecci et al. reported a homozygous mutation (p.R119C) in the *THPO* gene in a family with three affected children presenting at ages 3.5 years, 7 months, and 1.6 years with progression to pancytopenia. In all three affected children, treatment with the THPO-mimetic romiplostim led to remission [8]. In 2023, Capci et al. reported a child with a homozygous mutation (c.-323C>T) in the promoter region of the THPO gene who presented with severe amegakaryocytic thrombocytopenia with a low serum THPO level and a bone marrow examination showing normocellular marrow with severe hypoplasia of the megakaryocytes. The patient responded to treatment with the THPO receptor agonist eltrombopag [9].

In comparison with the above-reported cases, our case had a relatively late presentation at seven years of age with asymptomatic thrombocytopenia, in contrast to most of the reported cases of CAMT2, which have pancytopenia and are symptomatic at the time of diagnosis. Moreover, WES in our case showed a homozygous deletion of part of exon 6 of the *THPO* gene. To the best of our knowledge, this variant has not been described in the literature so far in individuals affected by THPO-related conditions.

THPO gene mutations cause CAMT2 to manifest in early infancy as thrombocytopenia with hypoplastic bone marrow and decreased or normal serum THPO levels, with later progression to bone marrow failure. Most patients present in infancy or early childhood and have a severe disease course, whereas some have later-onset and milder symptoms [1]. In such cases, bone marrow transplantation is ineffective, and treatment with thrombopoietin receptor agonists results in the restoration of trilineage hematopoiesis [6,7].

## Conclusions

This case report highlights the importance of considering CAMT2 as the cause of unexplained persistent thrombocytopenia in infancy even in late presentation. However, it is important to exclude common causes of thrombocytopenia initially such as inherited bone marrow failure syndromes. Based on the reported case, we highly recommend performing genetic testing in case of thrombocytopenia and absent or decreased megakaryocytes, as CAMT2 is mild and can respond to THPO receptor agonists while avoiding invasive, ineffective bone marrow transplantation for these THPO gene mutations.

## References

[1] (2024). OMIM. # 620481. Amegakaryocytıc thrombocytopenıa, congenıtal, 2; CAMT2. https://omim.org/entry/620481.

[2] Kaushansky K (2005). The molecular mechanisms that control thrombopoiesis. J Clin Invest.

[3] Yagi M, Ritchie KA, Sitnicka E, Storey C, Roth GJ, Bartelmez S (1999). Sustained ex vivo expansion of hematopoietic stem cells mediated by thrombopoietin. Proc Natl Acad Sci U S A.

[4] Cornish N, Aungraheeta MR, FitzGibbon L (2020). Monoallelic loss-of-function THPO variants cause heritable thrombocytopenia. Blood Adv.

[5] Feese MD, Tamada T, Kato Y (2004). Structure of the receptor-binding domain of human thrombopoietin determined by complexation with a neutralizing antibody fragment. Proc Natl Acad Sci U S A.

[6] Dasouki MJ, Rafi SK, Olm-Shipman AJ (2013). Exome sequencing reveals a thrombopoietin ligand mutation in a Micronesian family with autosomal recessive aplastic anemia. Blood.

[7] Seo A, Ben-Harosh M, Sirin M (2017). Bone marrow failure unresponsive to bone marrow transplant is caused by mutations in thrombopoietin. Blood.

[8] Pecci A, Ragab I, Bozzi V (2018). Thrombopoietin mutation in congenital amegakaryocytic thrombocytopenia treatable with romiplostim. EMBO Mol Med.

[9] Capaci V, Adam E, Bar-Joseph I, Faleschini M, Pecci A, Savoia A (2023). Defective binding of ETS1 and STAT4 due to a mutation in the promoter region of THPO as a novel mechanism of congenital amegakaryocytic thrombocytopenia. Haematologica.

